# Esthesioneuroblastoma chemotherapy and radiotherapy for extensive disease: a case report

**DOI:** 10.1186/1477-7819-9-118

**Published:** 2011-10-05

**Authors:** Seema Gupta, Nuzhat Husain, Sham Sundar

**Affiliations:** 1 Department of Radiotherapy, C.S.M. Medical University, Lucknow, Uttar Pradesh, India; 2 Department of Pathology, C.S.M. Medical University, Lucknow, Uttar Pradesh, India

**Keywords:** esthesioneuroblastoma, nasal cavity, pathology, immunohistochemistry, treatment

## Abstract

Malignant tumors of the nasal cavity are rare. We report the case of a 48 years old man who consulted us with a 2-year history of progressive nasal obstruction, occasional epistaxis, facial pain, and watering of the eyes. A diagnosis of olfactory neuroblastoma was established by histopathology and confirmed by immunohistochemistry. On staging, the mass was classified as a Kadish stage C tumor with extensive involvement of the nasal cavities, nasopharynx, paranasal sinuses and orbit. Endoscopic excision of the mass was done. Traditionally the mainstay of treatment in such locally advanced patients is craniofacial resection followed by adjuvant radiotherapy. Our patient was treated with limited surgery and chemoradiation. Patient is free of recurrence at a follow-up of 5 years. This case report demonstrates the potential efficacy of planned combined modality therapy, including limited surgery and early chemoradiation in the control of locally advanced olfactory neuroblastoma.

## Background

Malignant tumors of the nasal cavity are rare. Olfactory neuroblastomas also known as esthesioneuroblastomas (ENB) is a rare and aggressive malignant tumor accounting for only 6% of nasal cavity and paranasal sinus neoplasms and 0.3% of the upper aero digestive tract malignancies [1]. Treatment recommendations range from a minimally invasive approach to combined modality treatment including craniofacial resection and chemo radiotherapy [2].

### Case Presentation

In June 2004, a 48 year old male presented with progressive bilateral nasal obstruction, and bleeding through nostrils since 3 years associated with facial pain and blurred left eye vision from 6 months.

**Figure 1:**
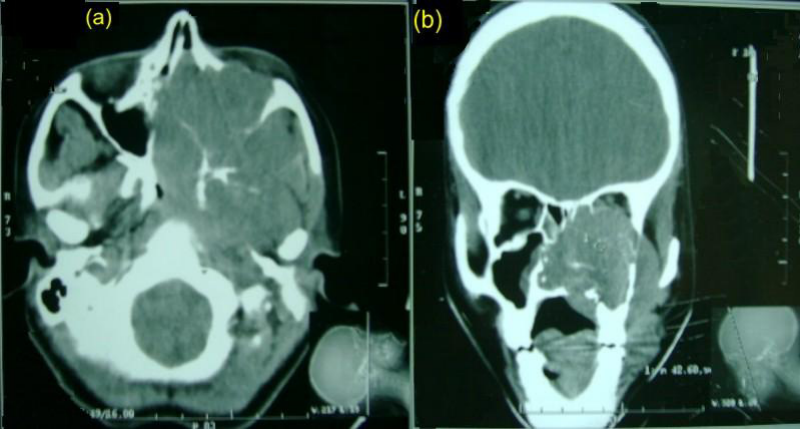
**Pre-treatment axial and coronal paranasal sinuses and orbit contrast-enhanced CT-Scan (a, b)**. Showing bulky soft-tissue mass in left maxilla, bilateral ethmoid sinuses, nasal cavity, pterygopalatine fossa, nasopharynx with destruction of medial and lateral wall of orbit.

Computed tomography dated- June 2004 (figure 1) showed large mass involving both nasal cavities, paranasal sinuses, nasopharynx, left pterygoid plates, left pterygopalatine fossa, hard palate, and orbit. There was no clinically or radiologically evident disease in neck, chest or abdomen. An endoscopically guided biopsy disclosed esthesioneuroblastoma.

**Figure 2:**
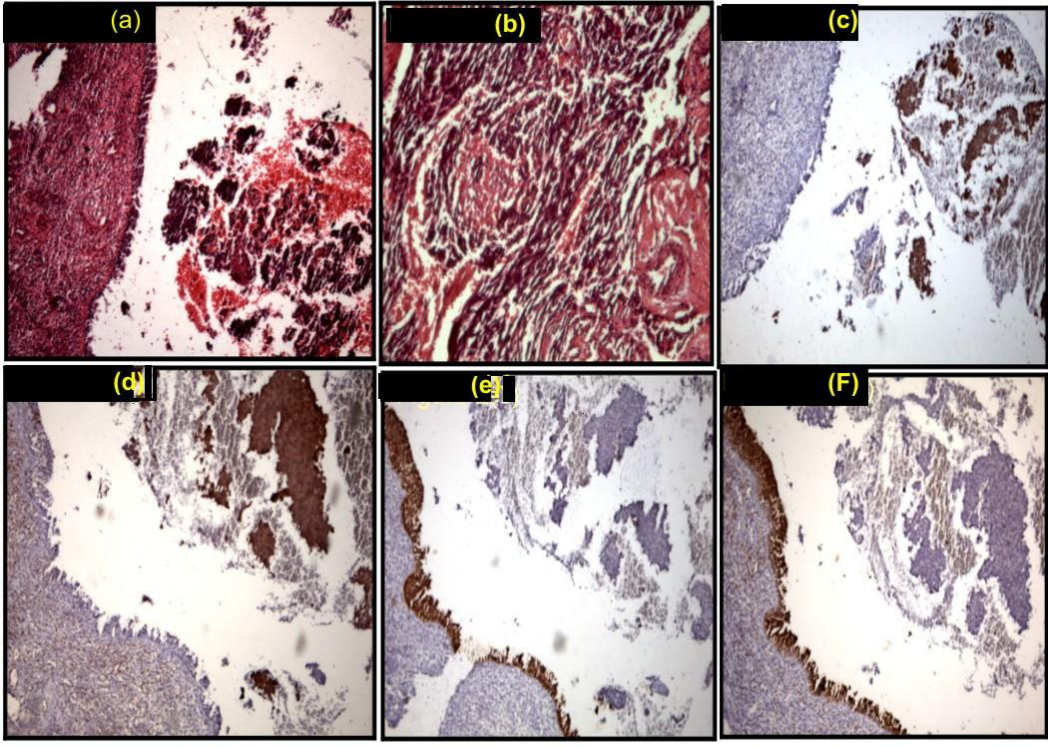
**Microphotograph showing small cells with rosettes (H & E × 125 × digital magnification) (a, b) neuron specific enolase positive in tumor cells(c, d), Cytokeratin and epithelial membrane antigen negative in tumor (DAB × 125 × digital magnification) (e, f)**.

Histopathology section showed an infiltrating round cell tumor with areas of necrosis and hemorrhage, partly ulcerating the overlying nasopharyngeal mature epithelium. Tumour cells showed nuclear molding with occasional rosettes (figure 2a and 2b).

Immunohistochemistry profile (figure 2c and 2d) showed positivity for neural marker namely neuron specific enolase. The tumor cells were negative for epithelial markers cytokeratin and epithelial membrane antigen (figure 2e and 2f) Hence a diagnosis of adult esthesioneuroblastoma of nasal cavity was made.

Patient was planned for upfront chemotherapy followed by radiotherapy in June 2004. Surgical excision in form of craniofacial surgical resection was not done due to non-availability of technical advances and surgical expertise. Patient received 6 cycles of cisplatin (20 mg/m^2 ^day1-5), etoposide (100 mg/m^2 ^day1-5) and bleomycin (30 mg day2, 9 and 16) at 3 weekly interval, followed by external beam radiotherapy. Radiotherapy was planned with one anterior and two lateral wedge pair fields with unequal weighting and treatment was delivered via Telecobalt Unit Theratron 780C ( AECL, Ottawa, Canada) with dose normalized at tumor center. Dose homogeneity requirement was 95-105% of the specified centrally absorbed dose, as mentioned in the ICRU 50-reference point. Two-dimensional computer planning was done on Radplan software (TSG Corporation, India). Patient received total dose of 56Gy in 28 fractions in 5 1/2 weeks.

**Figure 3:**
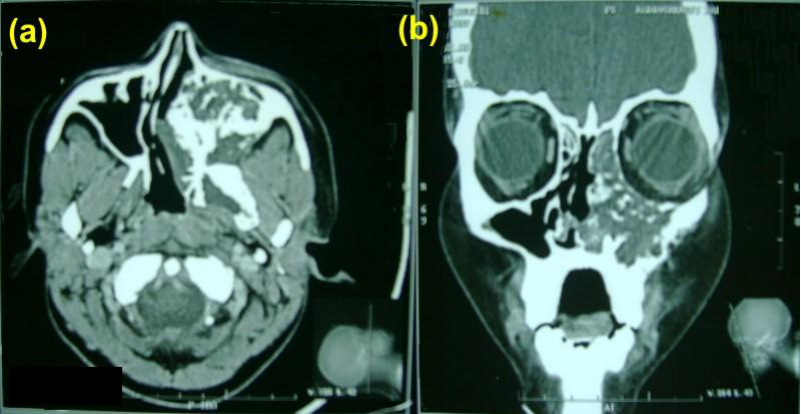
**Axial and coronal contrast-enhanced paranasal sinuses and orbit CT-scan after 6 cycles of chemotherapy and radiotherapy (a, b)**. showing marked reduction in the tumor, the lesion is limited to the left maxillary sinus. A partial tumor remission was considered.

**Figure 4:**
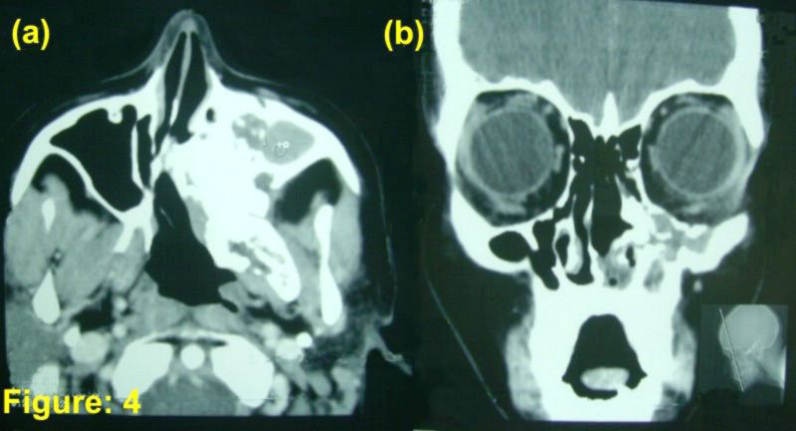
**Axial and coronal contrast-enhanced paranasal sinuses and orbit computed tomography scan after completion of treatment (a, b)**. The scan is showing post radiation changes most likely to be fibrosis.

Post radiation patient had significant improvement in symptoms but CT-Scan revealed residual disease (figure 3), therefore 4 more cycles of cisplatin (20 mg/m^2 ^day1-5) and etoposide (100 mg/m^2 ^day1-5) chemotherapy was given at 3 weekly interval. Treatment was completed in June 2005; subsequently our patient has been clinically asymptomatic through out the follow up period of 5 yrs. The latest C.T scan done in June 2010 (figure 4)showed evidence of post radiation changes most likely to be fibrosis in left maxillary antrum with marked new bone formation and no sign of disease.

## Discussion

Extension of primary tumor based on the Kadish staging system has been identified as the most important determinant of the treatment outcome [3,4].

Our patient who presented with locally advanced disease Kadish stage C without intracranial extension was treated with limited surgery combined with chemoradiotherapy which has shown good response and disease free survival of 5 years. Presently our patient is clinically and radiologically free of disease.

Craniofacial surgery is the mainstay of treatment in ENB regardless of stage and grade with the possible exception of distance metastasis followed by adjuvant radiation in high grade and locally advanced tumors. [5]. In unresectable ENB the experience with chemoradiation is lacking and is under-reported probably due to limited cases, but some of the latest series have shown promising results in nonsurgical approach [2].

Encouraged by these results we treated our patient who had locally advanced disease by limited surgery due to non-availability of technical advances and surgical expertise followed by chemo radiation, with excellent clinical outcome.

## Conclusion

Chemoradiation along with limited surgery can be considered as one of the treatment modalility for locally advanced ENB with promising results.

## Consent

Written informed consent was obtained from the patient for publication of this case report and accompanying images. A copy of the written consent is available for review by the Editor-in-Chief of this journal.

## Competing interests

The authors declare that they have no competing interests.

## Authors' contributions

SG carried out the history, examination, treatment and the provisional draft. NH carried out the histopathology and Immunohistochemistry. SS involved in the patient management and data collection. All authors read and approved the final manuscript.
